# Regulation of Amyloid Precursor Protein Processing by Serotonin Signaling

**DOI:** 10.1371/journal.pone.0087014

**Published:** 2014-01-21

**Authors:** Anna A. Pimenova, Amantha Thathiah, Bart De Strooper, Ina Tesseur

**Affiliations:** 1 KU Leuven Center for Human Genetics and Leuven research Institute for Neuroscience and Disease (LIND), Leuven, Belgium; 2 VIB Center for the Biology of Disease, Leuven, Belgium; Pasteur Institute of Lille, France

## Abstract

Proteolytic processing of the amyloid precursor protein (APP) by the β- and γ-secretases releases the amyloid-β peptide (Aβ), which deposits in senile plaques and contributes to the etiology of Alzheimer's disease (AD). The α-secretase cleaves APP in the Aβ peptide sequence to generate soluble APPα (sAPPα). Upregulation of α-secretase activity through the 5-hydroxytryptamine 4 (5-HT_4_) receptor has been shown to reduce Aβ production, amyloid plaque load and to improve cognitive impairment in transgenic mouse models of AD. Consequently, activation of 5-HT_4_ receptors following agonist stimulation is considered to be a therapeutic strategy for AD treatment; however, the signaling cascade involved in 5-HT_4_ receptor-stimulated proteolysis of APP remains to be determined. Here we used chemical and siRNA inhibition to identify the proteins which mediate 5-HT_4d_ receptor-stimulated α-secretase activity in the SH-SY5Y human neuronal cell line. We show that G protein and Src dependent activation of phospholipase C are required for α-secretase activity, while, unexpectedly, adenylyl cyclase and cAMP are not involved. Further elucidation of the signaling pathway indicates that inositol triphosphate phosphorylation and casein kinase 2 activation is also a prerequisite for α-secretase activity. Our findings provide a novel route to explore the treatment of AD through 5-HT_4_ receptor-induced α-secretase activation.

## Introduction

The most common form of dementia in elderly people is Alzheimer's disease (AD), which is pathologically characterized by progressive neuronal loss and deposition of the amyloid-β peptide (Aβ) in amyloid plaques. Current therapeutic targets are the β- and γ-secretases, which generate Aβ from amyloid precursor protein (APP). Several drugs inhibiting or modulating the activity of these secretases have failed in clinical trials due to severe side effects or to difficulty in delivery through the blood brain barrier [1]. APP is also cleaved by a third secretase called α-secretase. The α-secretase cleaves APP within the Aβ peptide sequence, producing a soluble APPα fragment (sAPPα), which precludes Aβ generation. Indeed, *in vivo* overexpression or upregulation of α-secretase activity in mice indicate that α-secretase activation leads to a decrease in Aβ production and a reduction in the amyloid plaque load in AD mouse models [2], [3]. These effects were accompanied by an improvement in the cognitive deficits, providing considerable support for modulation of α-secretase activity as a viable strategy in the fight against AD [2], [3].

To specifically target the non-amyloidogenic pathway of APP processing, a fundamental consideration would be to understand the mechanism of α-secretase activation and to determine the signaling cascade of kinases and second messengers that directly regulate α-secretase-mediated proteolysis of APP. These molecules can be directly targeted pharmacologically, but also indirectly via G protein-coupled receptors (GPCR), such as the muscarinic, glutamatergic and serotonergic receptors. In particular, the G protein coupled 5-hydroxytryptamine 4 (5-HT_4_) receptor is gaining considerable interest as a modulator of α-secretase activity due to its role in memory and learning and regulation of APP processing [4]. Activation of the 5-HT_4_ receptor leads to an increase in the population spike amplitude in the hippocampal CA1 region, and this effect persists in a transgenic mouse model of AD [5], [6], suggesting that 5-HT_4_ receptor-mediated signaling remains functional under these pathological conditions. On the other hand, agonist stimulation of the 5-HT_4_ receptor results in increased sAPPα secretion with a concomitant decrease in Aβ peptide levels in primary neuronal cultures and an alleviation of amyloid plaque load in AD mouse models [7]–[9]. Such amelioration of disease pathology is correlated with improvements in memory and learning in behavioral paradigms and scopolamine-induced models of cognitive deficit [10]–[12]. Additionally, an increase in acetylcholine release is observed after 5-HT_4_ receptor agonist application *in vivo* [13]. This could be a valuable property when considering 5-HT_4_ receptor agonists for AD treatment, which could complement the currently licensed therapy of cholinesterase inhibition for partial symptomatic relief [14].

Despite numerous reports on 5-HT_4_ receptor function in memory and learning and its effect on APP processing, the downstream signaling pathway responsible for this 5-HT_4_ receptor-mediated effect is still poorly understood. 5-HT_4_ receptor stimulation results in an accumulation of cAMP, a second messenger required for protein kinase A (PKA) and exchange protein activated by cAMP (Epac) activation. However, 5-HT_4_ receptor-mediated non-amyloidogenic processing of APP occurs independently of PKA activation, but can be regulated by Epac1 activation of Rac1 and Rap signaling in cell lines and primary neurons [15]. The 5-HT_4_ receptor is constitutively bound to the Src non-receptor tyrosine kinase, which is required for ERK activation [16]. In addition, 5-HT_4_ receptor stimulation in adrenocortical cells and cardiomyocytes results in an increase of calcium influx, which results in activation of voltage-gated calcium channels through PKA [17], [18]. It is unknown whether these latter pathways also contribute to α-secretase activation downstream of the 5-HT_4_ receptor. Altogether, these studies suggest a complicated picture of the downstream signaling pathways involved in 5-HT_4_ receptor stimulation and reveal the importance of delineation of the mechanism of 5-HT_4_ receptor-mediated APP proteolysis.

Finally, several metalloproteinases have been proposed as α-secretase; however, the identity of 5-HT_4_ receptor-induced α-secretase activity has not been fully addressed. The disintegrin and metalloprotease ADAM10, a major constitutive α-secretase of APP [19], [20], is a feasible candidate [21]. However, ADAM17 is more likely to be the inducible APP α-secretase based on studies which have investigated the regulated ectodomain shedding of other ADAM substrates after protein kinase C (PKC) activation [22]. In support of this is the observation that M1 receptor induced sAPPα release correlates with increased ADAM17 expression levels [23]. Nevertheless, additional metalloproteinases, such as meprin β and membrane-type matrix metalloproteinases, were shown to mediate α-cleavage of APP [24], [25].

Here, we specifically determined the intracellular signaling cascade involved in 5-HT_4d_ receptor stimulation and inducible α-secretase activity. We used human SH-SY5Y neuroblastoma cells to analyze APP processing for practical reasons and experimental consistency. Human SH-SY5Y cells can generate sustainable cells with characteristics that resemble the morphology and biochemistry of mature neurons [26]. We present evidence that the G protein-dependent pathway activating Src, phospholipase C (PLC) and casein kinase 2 (CK2) is responsible for the 5-HT_4d_ receptor-stimulated induction of α-secretase activity. Interestingly and in contrast to previous publications, we find that adenylyl cyclase (AC) and cAMP signaling are not required for 5-HT_4d_ receptor-mediated α-secretase activity [15]. Furthermore, we analyzed the reported α-secretases as putative mediators of the 5-HT_4d_ receptor effect on APP shedding using RNAi studies.

## Materials and Methods

### Reagents and Antibodies

Tissue culture reagents were purchased from Invitrogen. GF109203X, SQ22536, D609, ionomycin, 4,5,6,7-tetrabromo-1H-benzotriazole (TBB), GR113808, NF449, gallein and batimastat were obtained from Tocris. 5-hydroxytryptamine (5-HT), cholera toxin B (CTB) and chlorogenic acid (CGA) were from Sigma-Aldrich. Phorbol 12-myristate 13-acetate (PMA), 2,5-dideoxyadenosine (DDA), IP3K inhibitor, TAPI-1 and GM6001 were obtained from Calbiochem/VWR. Bosutinib was from Selleck. Prucalopride was kindly provided by Movetis (NV, Turnhout, Belgium). Table 1 summarizes the known potencies of the used agonists and antagonists. Following antibodies were purchased: CK2 α (H-286) from Santa Cruz, MMP-9 (G657) and ADAM9 (2099) from Cell Signaling, ADAM17 (T5442) and β-Actin (A5441) from Sigma-Aldrich. ADAM10 (B42) and APP (B63) antibodies were made in house and previously described [27]. Gα_s_ dominant negative construct was previously described (pcDNAI-Amp-Gα_s_DN, [28]).

**Table 1:** Overview of agonists, antagonists and inhibitors used to investigate the proteins contributing to the induction of sAPPα after 5-HT_4d_ receptor stimulation.

Compound	Target	Ag/antag/inh	Potency	Experimental system	Citation
Prucalopride	5-HT_4_	Ag	EC_50_ 10 nM	SH-SY5Y cells	[9]
5-HT	5-HT_4_	Ag	EC_50_ 1,1 nM	HEK293 cells	[34]
GR113808	5-HT_4_	Antag	K_i_ 0,31 nM	Mouse colliculi neurons	[63], [64]
Cholera toxin B (CTB)	Gα_s_	Inh	IC_50_ 100 ng/ml	L6 cells	[65], [66]
NF449	Gα_s_	Inh	IC_50_ 8 µM	*in vitro*	[67]
Gallein	Gβγ	Inh	IC_50_ 5 µM	HL60 cells	[68]
SQ 22536	Adenylyl cyclase	Inh	IC_50_ 1 µM	Human blood platelets	[69], [70]
2,5-dideoxyadenosine (DDA)	Adenylyl cyclase	Inh	IC_50_ 100 µM	*in vitro*	[71], [72]
Bosutinib	Src	Inh	IC_50_ 300 nM	MDA-MB-468 cells	[73], [74]
D609	Phospholipase C	Inh	K_i_ 6,4 µM	*in vitro*	[75], [76]
GF109203X	Protein kinase C	Inh	IC_50_≤5,8 µM	*in vitro*	[77], [78]
IP3K inhibitor	IP6K, IP3K	Inh	IC_50_ 18 µM	*in vitro*	[79]
Chlorogenic acid (CGA)	IPMK	Inh	IC_50_ 1,15 µM	*in vitro*	[80]
4,5,6,7-tetrabromo-1H-benzotriazole (TBB)	Casein kinase 2	Inh	IC_50_ 1,6 µM	*in vitro*	[81]–[83]
GM6001	MMP1, 2, 3, 8, 9; ADAM10 and 17	Inh	K_i_ 0,1–110 nM	*in vitro*	[84], [85]

Ag = agonist; antag = antagonist; inh = inhibitor.

### Inhibitor treatment and soluble APP analysis (SEAP assay)

SH-SY5Y human neuroblastoma cells (CRL-2266, ATCC) were cultured in DMEM/F12 supplemented with 10% fetal bovine serum (FBS). For analysis of soluble APP secretion, a mix of 1,5 µg plasmid encoding human wild type APP695 linked to Alkaline Phosphatase (AP-APP) at the N-terminus (pEAK12-AP-APP, [29]), 1,35 µg of 5-HT_4d_ receptor isoform in pcDNA3.1 (pcDNA3.1-5-HT_4d_, kindly provided by Joris De Mayer and Jan Schuurkes, Movetis, Turnhout, Belgium) and 0,15 µg of GFP (pmaxFP-Green-N, Amaxa) was prepared in OPTI-MEM and combined with 20 µl of Lipofectamine 2000 (Invitrogen). After 20 minutes incubation at room temperature transfection mix was combined with a trypsinized cell suspension in growth medium containing 10% FBS. After another 15 minutes incubation at room temperature cells were seeded in a 96-well plate at 80.000 cells/well. The next day medium was changed to DMEM/F12 supplemented with 5% dialyzed FBS (10,000 molecular weight cutoff), which is devoid of serotonin otherwise present in undialyzed FBS that causes 5-HT_4_ receptor desensitization. After three days, cells were washed and incubated in serum free medium (SFM) for another 24 hours. Next cells were stimulated with 1 µM of the following compounds: prucalopride, 5-HT, PMA and GR113808 in SFM for 24 hours and the conditioned medium was analyzed for secreted AP (SEAP) activity with Great EscAPe SEAP Chemiluminescence Kit 2.0 (Clontech) according to manufacturer's instructions. Luminescence was measured with the EnVision® multilabel reader (PerkinElmer). For signaling studies dilution curves of inhibitory compounds were made in combination with induction by 1 µM prucalopride or 5-HT. DMSO incubation was used as a control in all experiments carried out. The ratio of individual luminescence counts from the tested conditions to the mean value of DMSO treated cells was plotted as SEAP fold induction. Cells were used for the MTS proliferation assay (CellTiter 96® AQ_ueous_ Non-Radioactive Cell Proliferation Assay) and the LDH cytotoxicity assay (CytoTox 96® Non-Radioactive Cytotoxicity Assay) according to manufacturer's instructions (Promega). Compound dilution curves were performed in the range of the reported effective concentrations (Table 1) and working concentrations were determined in the SEAP assay as those giving significant inhibition of 5-HT_4d_ receptor-stimulated sAPPα secretion. MTS and LDH assays were used to define working concentrations of the different compounds that were non-toxic to the cells.

### cAMP assay

cAMP levels were assessed using the Alphascreen® cAMP assay kit (PerkinElmer Life Sciences). 2,88·10^6^ or 1·10^6^ SH-SY5Y cells were seeded in T75 or T25 flasks, respectively. Adherent cells were transfected after 4 hours with Lipofectamine and Plus reagent (Invitrogen) according to the manufacturer's instructions. A mix of 7,5 µg pEAK12-AP-APP, 6 µg pcDNA3.1-5-HT_4d_ and 1,5 µg pmaxFP-Green-N plasmids (ratio of 5∶4∶1) was used for transfection in T75 flasks. A mix of 1,25 µg pEAK12-AP-APP, 0,65 µg pcDNA3.1-5-HT_4d_ receptor and 2,6 µg pcDNAI-Amp-Gα_s_DN or pcDNA3.1 as an empty vector control (ratio of 2∶1∶4) was used for transfection in T25 flasks. 3 hours later transfection mixes were replaced with growth medium for 16 hours and cells were treated with medium supplemented with 5% dialyzed FBS and SFM as described under “inhibitor treatment and soluble APP analysis”. Then cells were gently dissociated with Versene solution (Invitrogen) to obtain a single cell suspension. Next cells were counted to determine the exact cell number. Equal numbers of cells were combined with the acceptor beads coupled to an anti-cAMP antibody and biotinylated cAMP, both provided in the Alphascreen® cAMP assay kit (PerkinElmer Life Sciences), and a serial dilution of compound. After incubating the cells for one hour, streptavidin-donor beads were added and the cells were permeabilized with 0,3% Tween-20 for 30 minutes, which released intracellular cAMP. The assay is based on competition between endogenously produced cAMP by the stimulated cells and exogenously added biotinylated cAMP. The electron transfer between donor and acceptor beads was measured with the EnVision® multilabel reader (PerkinElmer). DMSO was diluted to a final concentration of 0,1% and kept equal in all samples to avoid differential effects of different DMSO concentrations on the cells. cAMP concentrations were determined using a standard curve.

### Calcium measurements

Calcium imaging was assessed using the Fluo-4 NW calcium assay kit (Invitrogen). SH-SY5Y cells were transfected with pEAK12-AP-APP, pcDNA3.1-5-HT_4d_ receptor and pmaxFP-Green-N in Optilux black wall clear bottom plates (BD Biosciences) and treated as described under “inhibitor treatment and soluble APP analysis”. Next cells were loaded with Fluo-4 NW dye mix according to manufacturer's instructions. Binding with calcium ions increases fluorescence of the dye. Baseline fluorescence of the dye was recorded at the steady state, while stimulated calcium release was assessed after automated addition of the compounds at different time points using IN Cell Analyzer 2000 (GE Healthcare). Calcium images were analyzed using the “Plot Z-axis Profile” function of ImageJ (NIH). Data are presented as a ratio of fluorescence intensity of Fluo-4 NW at any given time to baseline fluorescence (F/F_0_).

### Construction of mutated cDNA

Mutations in the cDNA of the 5-HT_4d_ receptor were introduced using the QuickChange II XL site-directed mutagenesis kit from Stratagene. All vector modifications were validated with sequencing using BigDye® Terminator v3.1 Cycle Sequencing and the ABI Prism® 3100 Genetic Analyzer (Applied Biosystems). Obtained data were analyzed with the Sequence Scanner program and LALIGN tool from ch.embnet.org.

### siRNA-mediated knockdown and immunoblotting

Knockdown of the proteins of interest was performed 4 hours after SH-SY5Y cells were transfected with pEAK12-AP-APP, pcDNA3.1-5-HT_4d_ receptor and pmax-FP-Green-N plasmids. Half of the medium was replaced with transfection mix containing 3 nM target protein siRNA and Lipofectamine RNAiMAX (Invitrogen) and left on the cells overnight. Next we proceeded with the protocol as described under “inhibitor treatment and soluble APP analysis”. The following siRNAs were used: Stealth RNAi™ siRNAs were used for GNAS HSS104240, GNAQ HSS104237, GNA13 HSS173827, PLCG1 HSS108094, CSNK2A1 HSS175396, ADAM9 HSS189548, MMP9 HSS181135 and BLOCK-iT™ Alexa Fluor® Red Fluorescent Oligo as a control (Invitrogen). The siGENOME SMARTpool was used for ADAM10 and siGENOME Non-Targeting siRNA Pool #1 as a control (Dharmacon). The FlexiTube GeneSolution GS6868 SI02664501 was used for ADAM17 and AllStars Negative Control siRNA as a control (QIAgen). Conditioned medium was collected to measure SEAP activity. For detection of Gα_s_, Gα_q_, Gα_13_, CK2, ADAM9, 10, APP and β-Actin, cells were lysed in RIPA buffer (50 mM Tris-HCl pH 7.4, 150 mM NaCl, 1% NP-40, 0.5% Sodiumdeoxycholate, 0.1% SDS and Complete protease inhibitor tablets (Roche Applied Science)). For detection of MMP9, conditioned medium was cleared from cell debris by centrifugation at 1500 rpm and concentrated with Ultracel-50 centrifugal filter unit (Millipore) according to manufacturer's instructions. For detection of ADAM17, cells were homogenized in 50 mM Tris-HCl pH 8.0 and 150 mM NaCl, 1 µM batimastat and Complete protease inhibitor tablets and centrifuged at 100.000 g for 1 hour at 4°C. Pellets were resuspended in RIPA buffer supplemented with 1 µM batimastat and centrifuged at 100.000 g for 1 hour at 4°C and supernatants containing the membrane fraction were collected. Protein concentrations were determined in each preparation using the Bradford assay (Bio-Rad). Equal amounts of protein were separated with SDS-PAGE in Novex Bis-Tris gels (Invitrogen), transferred to nitrocellulose membranes (Whatman), blocked and probed with antibodies in 3% milk plus 0,1% Tween-20/TBS buffer. Secondary antibody staining was detected with the Renaissance chemiluminescence kit (Perkin Elmer). β-Actin staining was used as a loading control.

### Statistical analysis

Differences between multiple means of data with parametric distribution were assessed by ANOVA followed by Tukey-Kramer or Dunnet's post-hoc tests. All experiments were repeated three times. All statistical analysis was performed with GraphPad Prism 5 (GraphPad Software). Optical density of specific immunobands on western blot was quantified using the 1D densitometry package of Aida Image Analyzer v4.27.039.

## Results

### 5-HT_4d_ receptor-stimulated APP shedding requires G protein signaling and is independent of β-arrestin recruitment

**Figure 1:**
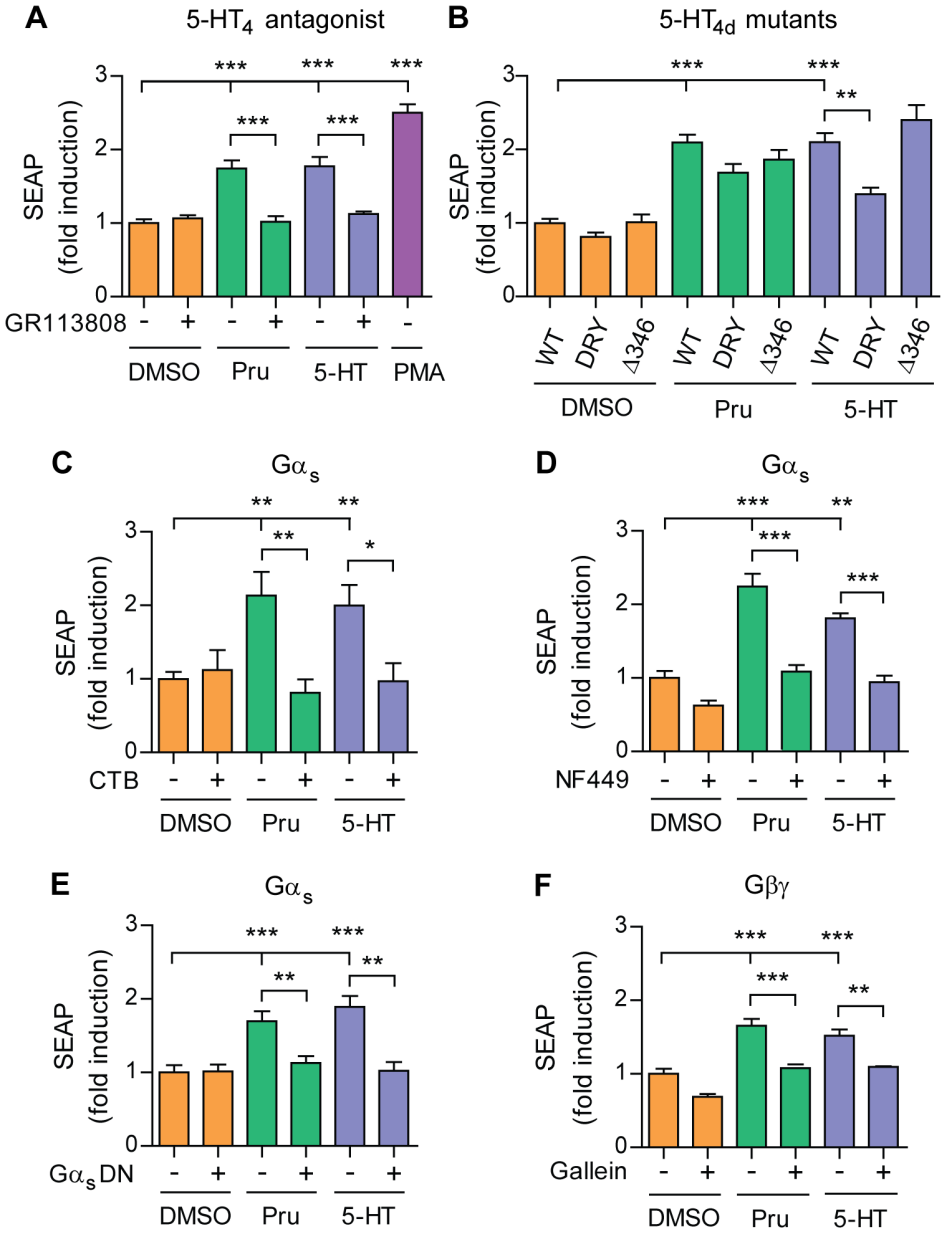
5-HT_4d_ receptor-stimulated APP shedding requires G protein signaling and is independent of β-arrestin recruitment. (A) Prucalopride induced sAPPα secretion in SH-SY5Y human neuroblastoma cells is specific for the 5-HT_4_ receptor. SH-SY5Y cells, transfected with pEAK12-AP-APP and pcDNA3.1-5-HT_4d_, were treated with 1 µM prucalopride and 5-HT (5-HT_4_ receptor agonists) in the absence or presence of 1 µM GR113808 (5-HT_4_ receptor antagonist) or PMA and secretion of sAPPα was analyzed via measuring SEAP. (B) SEAP levels were measured in supernatants of SH-SY5Y cells, transfected with pEAK12-AP-APP and pcDNA3.1-5-HT_4d_ (WT), pcDNA3.1-5-HT_4d_DRY117/118AAY (DRY) or pcDNA3.1-5-HT_4d_Δ346 (Δ346) mutants and stimulated with 1 µM prucalopride or 5-HT. (C), (D) and (F) SEAP levels were measured in supernatants of SH-SY5Y cells, transfected with pEAK12-AP-APP and pcDNA3.1-5-HT_4d_ and treated with 1 µM prucalopride or 5-HT in the absence or presence of 100 µM CTB (Gα_s_ inhibitor) (C), 100 µM NF449 (Gα_s_ inhibitor) (D) or 100 µM gallein (Gβγ inhibitor) (F). (E) SEAP levels were measured in SH-SY5Y cells, transfected with pEAK12-AP-APP, pcDNA3.1-5-HT_4d_ and pcDNAI-Amp-Gα_s_DN or pcDNA3.1 at a ratio of 2∶1∶4, respectively, and treated with 1 µM prucalopride or 5-HT. Values shown are mean ± SEM of 6 individual wells and were normalized to vehicle control. * *P*<0.05, ** *P*<0.01, *** *P*<0.001, one-way ANOVA with Tukey-Kramer or Dunnet's post-hoc test.

In agreement with previous reports which show that activation of the 5-HT_4d_ receptor induces APP shedding in CHO cells [7], we find a significant upregulation of SEAP (secreted alkaline phosphatase) activity in SH-SY5Y human neuroblastoma cells transiently transfected with human 5-HT_4d_ (pcDNA3.1-5-HT_4d_) and human wild type APP695 coupled to SEAP (pEAK12-AP-APP) following treatment with the 5-HT_4_ receptor agonists prucalopride or 5-HT. Co-treatment with the 5-HT_4_ receptor antagonist GR113808 abolishes the increase in AP-APP shedding, demonstrating the specificity of the effect (Figure 1A). Using an alphaLISA specific for sAPPα, we previously demonstrated that the increase in SEAP activity reflects an increase in sAPPα release [9].

Signaling pathway activation down-stream of the 5-HT_4_ receptor is mediated through assembly and activation of a heterotrimeric G protein complex. Receptor phosphorylation by GPCR-related kinases (GRKs) limits G protein-mediated signaling and facilitates recruitment of β-arrestins, which mediate receptor internalization and turnover and provide a scaffold for the initiation of signals to several kinases [30]. We used 5-HT_4d_ receptor mutants deficient in either G protein or β-arrestin signaling to distinguish which pathway leads to increased sAPPα secretion upon receptor stimulation. We introduced mutations in the DRY conserved motif, which interrupt coupling to G proteins and prohibit the G protein complex from acquiring an active GTP bound state [31], [32]. Expression of the alanine substitution DRY mutant of the 5-HT_4d_ receptor (pcDNA3.1-5-HT_4d_DRY117/118AAY) in pEAK12-AP-APP transfected cells resulted in significant downregulation of sAPPα secretion after 5-HT treatment, indicating the putative involvement of G protein signal transduction in 5-HT_4d_ receptor-stimulated release of sAPPα (Figure 1B). Recruitment of β-arrestins to the receptor requires phosphorylation of the C terminus, allowing further internalization and possible signal transduction through the scaffolding of down-stream kinases. We generated a 5-HT_4d_ receptor mutant truncated at amino acid 346 in the C terminus (pcDNA3.1-5-HT_4d_Δ346), which lacks a conserved sequence of serine and threonine residues required for association of β-arrestins with the receptor after phosphorylation by GRKs. Expression of this mutant in pEAK12-AP-APP transfected cells maintained stimulated induction of sAPPα secretion after prucalopride and 5-HT treatment (Figure 1B). These data suggest that β-arrestins do not contribute to 5-HT_4d_ receptor-induced APP shedding.

In order to confirm that G proteins participate in α-secretase induction, we co-treated pEAK12-AP-APP transfected cells with inhibitors of Gα_s_, i.e. CTB and NF449. Inhibition of Gα_s_ signaling indeed resulted in significant decrease of induced sAPPα secretion (Figures 1C and D). We also tested a Gα_s_DN mutant, which abolishes all GPCR-mediated G protein-dependent signaling [28]. We found that co-transfection of pcDNAI-Amp-Gα_s_DN with pEAK12-AP-APP and pcDNA3.1-5HT_4d_ inhibited induced sAPPα secretion after prucalopride and 5-HT treatment (Figure 1E), indicating that Gα_s_ activation is involved in 5-HT_4d_ receptor-stimulated α-secretase activity.

The 5-HT_4_ receptors promiscuously activate several G proteins, i.e. Gα_s_, Gα_q_ and Gα_13_, leading to distinct second messenger generation [33]–[35]. We wondered which G protein subunit is specifically responsible for the effect on sAPPα secretion. Therefore, we performed RNAi mediated knock-down of Gα_s_, Gα_q_ and Gα_13_ in SH-SY5Y cells and analyzed sAPPα secretion upon 5-HT_4d_ receptor stimulation (Figure S1A). Knock-down efficiency and specificity of siRNA oligonucleotides was confirmed by western blotting (Figures S1B and C). Surprisingly, our data show that single knock-down of each individual Gα subunit equally abolishes 5-HT_4d_ receptor-mediated sAPPα secretion (Figure S1A), suggesting that sAPPα release can be mediated through Gα_s_, Gα_q_ and Gα_13_. Such an effect could be explained if there is a requirement for the functional activation of the Gβγ subunits. We used gallein to inhibit Gβγ signaling and found that co-treatment of pEAK12-AP-APP transfected cells with this inhibitor and prucalopride or 5-HT abolished induction of sAPPα secretion (Figure 1F). Altogether, these studies suggest that Gα and Gβγ activation is required for 5-HT_4d_ receptor-stimulated sAPPα release.

### 5-HT_4d_ receptor-stimulated APP shedding does not involve activation of adenylyl cyclase and cAMP

**Figure 2:**
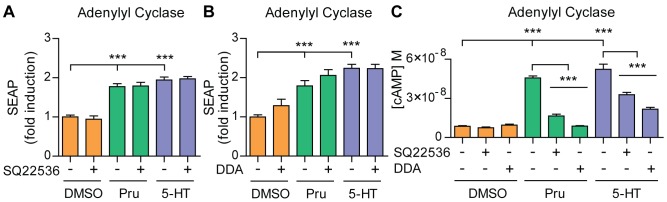
5-HT_4d_ receptor-stimulated APP shedding does not require accumulation of cAMP. (A) and (B) SH-SY5Y cells, transfected with pEAK12-AP-APP and pcDNA3.1-5-HT_4d_, were treated with 1 µM prucalopride or 5-HT (5-HT_4_ receptor agonists) in the absence or presence of 4 µM SQ22536 (AC inhibitor) (A) or 100 µM DDA (AC inhibitor) (B) and secretion of sAPPα was analyzed via measuring SEAP. (C) Concentration of cAMP was measured in SH-SY5Y cells, transfected with pEAK12-AP-APP, pcDNA3.1-5-HT_4d_ and pmax-FP-Green-N at a ratio of 5∶4∶1, were treated with 1 µM prucalopride or 5-HT in the absence or presence of 4 µM SQ22536 or 100 µM DDA. Values shown are mean ± SEM of 6 individual wells and were normalized to vehicle control. *** *P*<0.001, one-way ANOVA with Tukey-Kramer's post-hoc test.

Gα_s_ and cAMP mediate canonical signaling of 5-HT_4_ receptors [33]. Therefore, we sought to determine whether, in SH-SY5Y cells, accumulation of cAMP is also necessary for α-secretase activity as previously described for CHO cells [15]. We used the adenylyl cyclase inhibitors SQ22536 and DDA, which potently inhibit increases in cAMP levels (Figure 2C). Interestingly, we found that these inhibitors do not affect prucalopride or 5-HT-stimulated sAPPα release in SH-SY5Y cells (Figures 2A and B). These results suggest that activation of adenylyl cyclase and accumulation of cAMP is not required for 5-HT_4d_ receptor-stimulated APP shedding.

### 5-HT_4d_ receptor-stimulated APP shedding requires Src and phospholipase C

**Figure 3:**
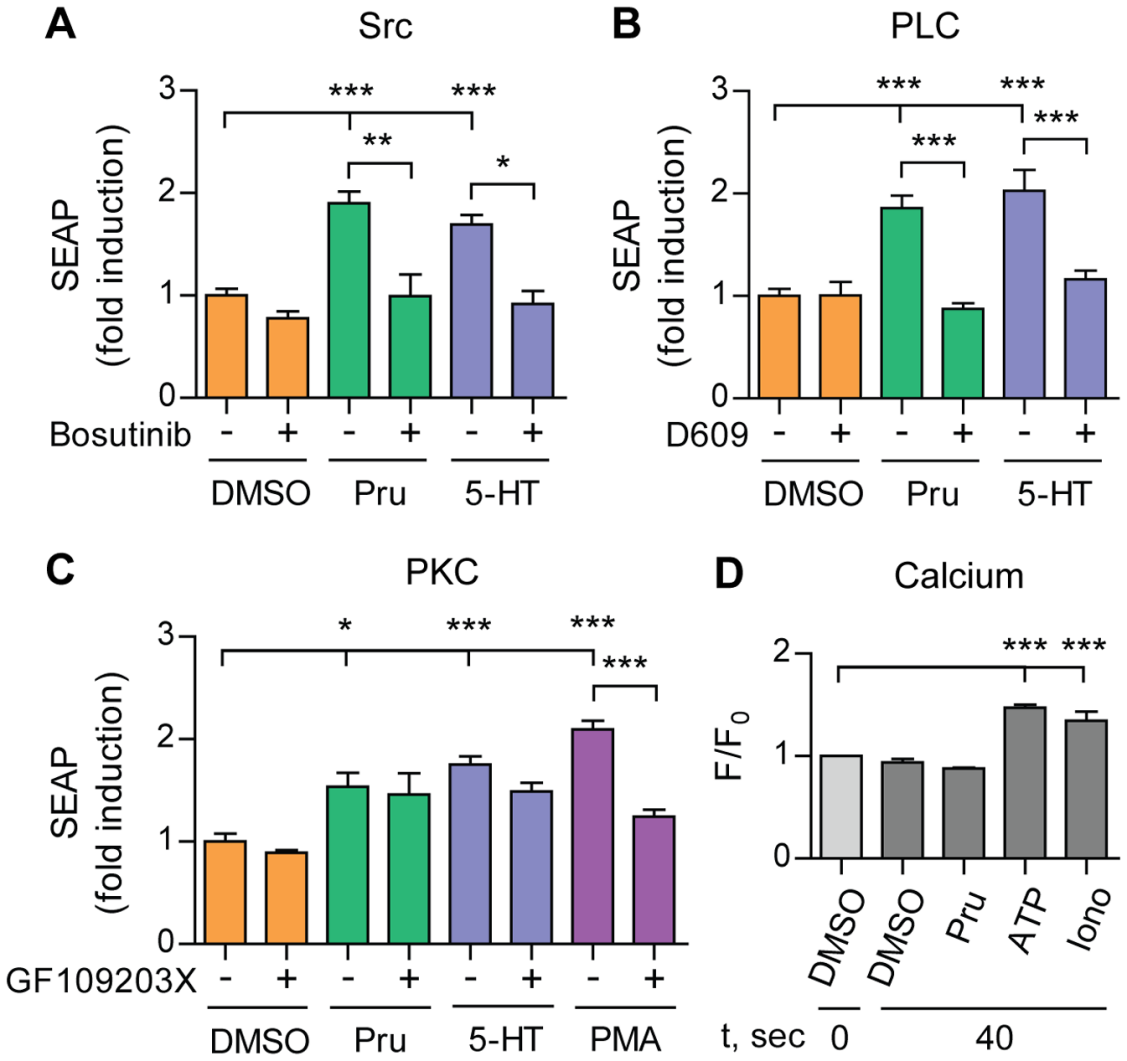
5-HT_4d_ receptor-stimulated APP shedding requires Src and phospholipase C, but not PKC or calcium. (A), (B) and (C) SH-SY5Y cells, transfected with pEAK12-AP-APP and pcDNA3.1-5-HT_4d_, were treated with 1 µM prucalopride or 5-HT (5-HT_4_ receptor agonists) and PMA in the absence or presence of 50 µM Bosutinib (Src inhibitor) (A), 30 µM D609 (PLC inhibitor) (B) or 2 µM GF109203X (PKC inhibitor) (C) and secretion of sAPPα was analyzed via measuring SEAP. Values shown are mean ± SEM of 6 individual wells and were normalized towards vehicle control. (D) SH-SY5Y cells, transfected with pEAK12-AP-APP and pcDNA3.1-5-HT_4d_, were loaded with Fluo-4 NW mix and fluorescence of the calcium-sensitive dye in each well was recorded at the baseline (F_0_) and after stimulation with 1 µM prucalopride, 30 µM ATP (purinergic ionotropic receptors agonist), 20 µM Ionomycin (calcium ionophore) or DMSO (F). Calcium response shown is a ratio of maximum fluorescence intensity at 40 sec to baseline fluorescence (F/F_0_). Values shown are mean ± SEM of 2 individual wells and were normalized to vehicle control. * *P*<0.05, ** *P*<0.01, *** *P*<0.001, one-way ANOVA with Tukey-Kramer's post-hoc test.

Given that 5-HT_4d_ receptor-stimulated APP shedding does not require an elevation in cAMP levels, we sought to determine whether generation of inositol triphosphate (IP3) is involved in 5-HT_4d_ receptor-stimulated sAPPα release. This second messenger is produced by PLC and can be activated either directly down-stream of Gα_q_ and Gβγ or through the Src non-receptor tyrosine kinase (reviewed in [36]). To analyze the contribution of PLC and Src, we co-treated pEAK12-AP-APP and pcDNA3.1-5-HT_4d_ transfected SH-SY5Y cells with the Src inhibitor Bosutinib or the PLC inhibitor D609 and 5-HT_4_ receptor agonists. In both cases, we observed that APP shedding was abolished compared to control treatment (Figures 3A and B).

PLC cleaves phosphatidylinositol 4,5-bisphosphate into IP3 and diacylglycerol, resulting in mobilization of intracellular calcium and activation of several downstream effector proteins including PKC [37]. In addition, several studies suggest that calcium and PKC signaling can activate α-secretase shedding of APP [38], [39]. Co-treatment of transiently transfected SH-SY5Y cells with the PKC inhibitor GF109203X did not induce sAPPα secretion after 5-HT_4d_ receptor stimulation. In contrast, direct activation of PKC with PMA induced sAPPα, but this induction was inhibited with GF109203X showing that the inhibitor was functional (Figure 3C). Similarly, prucalopride did not significantly alter extracellular calcium influx in SH-SY5Y cells, in contrast to ionomycin and ATP; two positive controls that prove assay functionality (Figure 3D). Taken together these data suggest that Src and PLC, but not PKC or calcium signaling, contribute to 5-HT_4d_ receptor-induced APP shedding.

### 5-HT_4d_ receptor-stimulated APP shedding requires inositol polyphosphates and casein kinase 2

**Figure 4:**
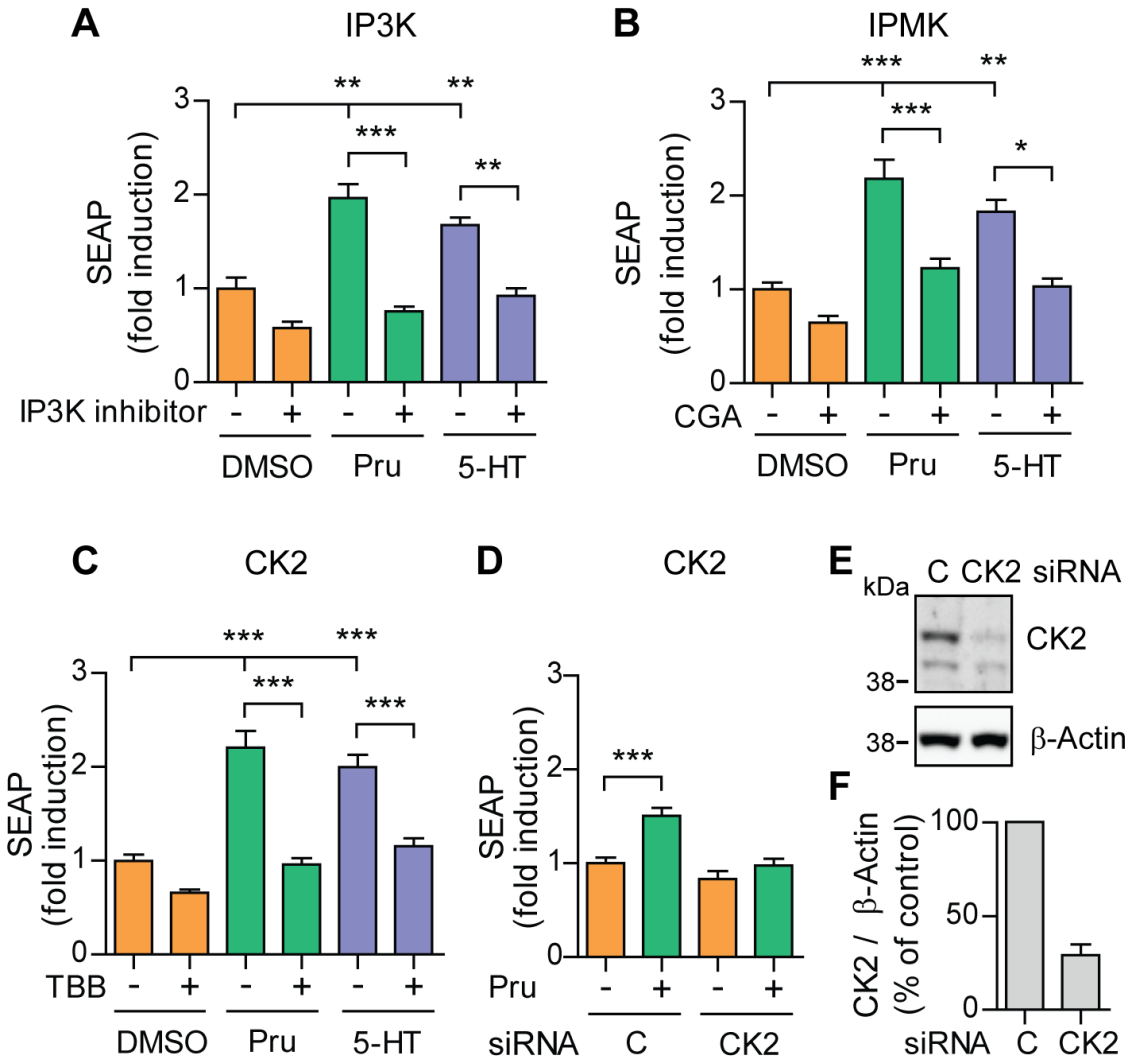
5-HT_4d_ receptor-stimulated APP shedding requires inositol polyphosphates and casein kinase 2. (A), (B) and (C) SH-SY5Y cells, transfected with pEAK12-AP-APP and pcDNA3.1-5-HT_4d_, were treated with 1 µM prucalopride or 5-HT (5-HT_4_ receptor agonists) in the absence or presence of 20 µM IP3K inhibitor (A), 80 µM CGA (IPMK inhibitor) (B) or 2.5 µM TBB (CK2 inhibitor) (C) and secretion of sAPPα was analyzed via measuring SEAP. (D) SEAP levels were measured in supernatants of SH-SY5Y cells, co-transfected with pEAK12-AP-APP, pcDNA3.1-5-HT_4d_ and 3 nM siRNA for knock-down of CK2 and treated with 1 µM prucalopride. (E) Cell lysates of (D) were analyzed for CK2 expression levels by western blotting. (F) Quantification of experiments in (E). Values shown are mean ± SEM of 6 individual wells and were normalized to vehicle control. * *P*<0.05, ** *P*<0.01, *** *P*<0.001, one-way ANOVA with Tukey-Kramer's post-hoc test.

IP3 can be further phosphorylated by inositol 1,4,5-triphosphate 3-kinase (IP3K) and inositol polyphosphate multikinase (IPMK) to generate inositol polyphosphates (IP4, IP5 and IP6). These molecules recently emerged as versatile second messengers with an increasing number of cellular functions [40]. We tested the IP3K inhibitor and the IPMK inhibitor CGA in transfected SH-SY5Y cells and found that prucalopride or 5-HT-stimulated α-secretase activity depends on the generation of these inositol polyphosphates (Figures 4A and B). The reported literature suggests that IP4 and/or IP6 can activate CK2 *in vitro* [41]. In cells, Wnt3a can induce IP5 generation which then activates CK2 [42]. Inhibition of CK2 activity with TBB in pcDNA3.1-5-HT_4d_ receptor and pEAK12-AP-APP expressing SH-SY5Y cells stimulated with prucalopride or 5-HT led to a decrease in sAPPα down to baseline levels (Figure 4C), suggesting that CK2 is involved in 5-HT_4d_ receptor-stimulated APP shedding. In addition, we found that co-transfection of CK2 siRNA completely abolished stimulated APP shedding in SH-SY5Y cells treated with prucalopride or 5-HT (Figures 4D, E and F). These results demonstrate that 5-HT_4d_ receptor-stimulated APP shedding requires inositol polyphosphates and CK2.

### ADAM9, ADAM10, ADAM17 and MMP9 are not responsible for 5-HT_4d_ receptor-stimulated α-secretase activity

**Figure 5:**
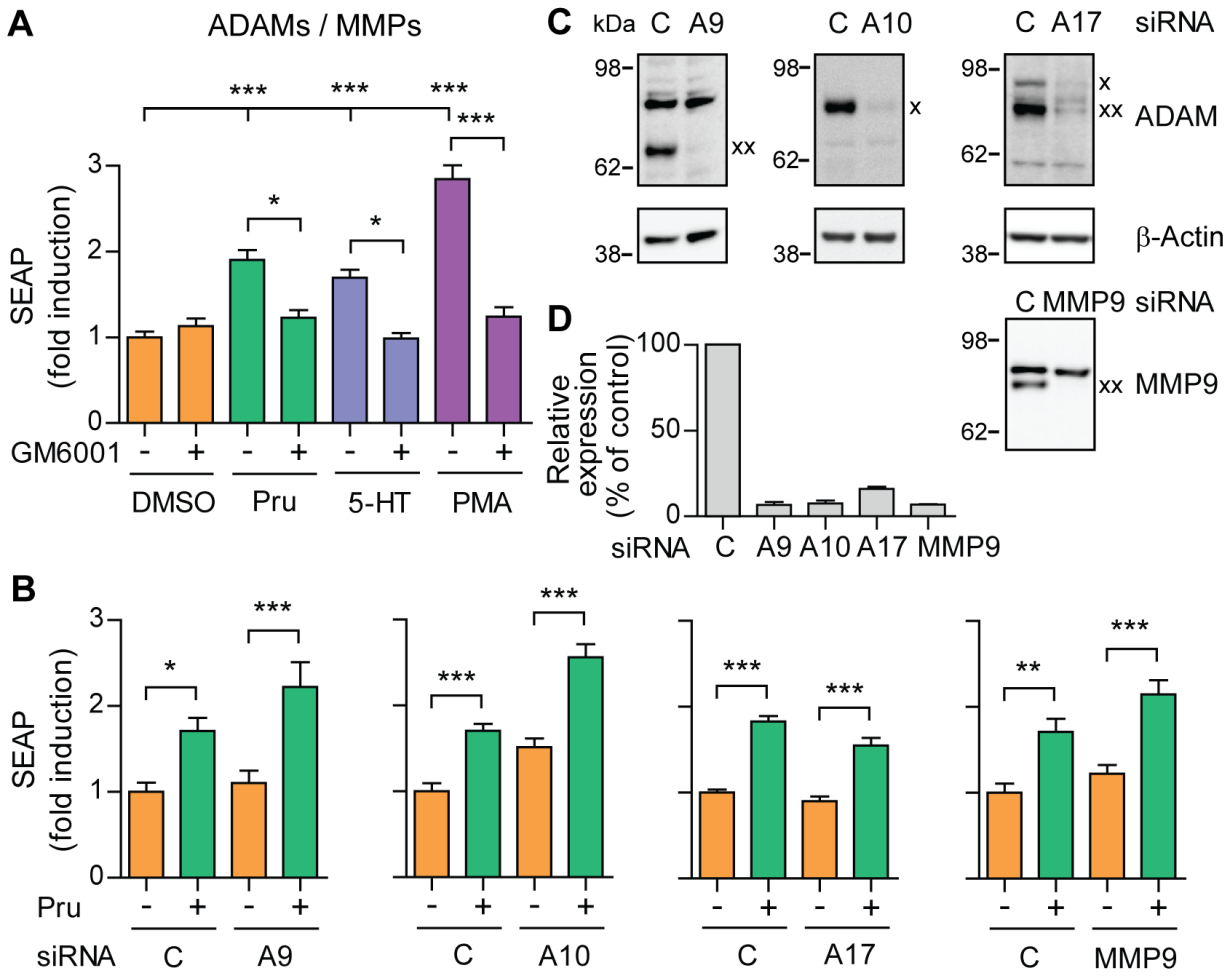
Single knock-down of ADAM9, ADAM10, ADAM17 and MMP9 does not affect 5-HT_4d_ receptor-stimulated APP shedding. (A) SH-SY5Y cells, transfected with pEAK12-AP-APP and pcDNA3.1-5-HT_4d_, were treated with 1 µM prucalopride or 5-HT (5-HT_4_ receptor agonists) in the absence or presence of 80 µM GM6001 (metalloproteinases inhibitor) and secretion of sAPPα was analyzed via measuring SEAP. (B) SEAP levels were measured in supernatants of SH-SY5Y cells, co-transfected with pEAK12-AP-APP, pcDNA3.1-5-HT_4d_ and 3 nM siRNA for knock-down of ADAM9 (A9), ADAM10 (A10), ADAM17 (A17) and MMP9 and treated with 1 µM prucalopride. (C) Cell lysates of (B) were analyzed for protein expression of ADAM9, 10, 17 and MMP9 by western blotting. The ADAM10 and ADAM17 immature precursor proteins are indicated by an x, whereas the mature catalytically active forms are indicated by an xx for ADAM9, 17 and MMP9. The immature ADAM9 and the mature ADAM10 proteins were not visible. (D) Quantification of experiments in (C). Values shown are mean ± SEM of 6 individual wells and were normalized to vehicle control. * *P*<0.05, ** *P*<0.01, *** *P*<0.001, one-way ANOVA with Tukey-Kramer's post-hoc test.

Several enzymes of the ADAM and MMP family, such as ADAM9, 10, 17 and MMP9, are suggested candidate proteins responsible for inducible shedding of APP (reviewed in [43]). To determine the relative contribution of the metalloproteinases in 5-HT_4d_ receptor-stimulated sAPPα release, we first analyzed expression levels of ADAM9, 10, 17 and MMP9 in SH-SY5Y cells. Expression of ADAM9, 10, 17 and MMP9 was not changed after prucalopride treatment of pEAK12-AP-APP transfected SH-SY5Y cells (Figures S3A and B). To test whether a metalloproteinase would be responsible for induced α-secretase activity, we treated the cells with non-toxic concentrations of the broad spectrum metalloproteinase inhibitor GM6001 (dose response curve shown in Figure S4). Treatment with GM6001 abolished induction of sAPPα secretion (Figure 5A), confirming that a metalloproteinase is indeed responsible for 5-HT_4d_ receptor-stimulated sAPPα release. To identify the 5-HT_4d_ receptor-stimulated α-secretase, we performed RNAi knock-down of the candidate α-secretases. We found that induction of sAPPα release was preserved after prucalopride treatment and single knock-down of ADAM9, 10, 17 or MMP9 (Figure 5B). The efficiency of the downregulation was between 85–95% as documented by western blot analysis (Figures 5C and D). These data suggest that ADAM9, 10, 17 or MMP9 are not responsible for 5-HT_4d_ receptor-mediated inducible α-secretase activity in SH-SY5Y cells. We also analyzed constitutive sAPPα secretion upon ADAM10 knock-down in non-treated cells and confirmed that ADAM10 acts as the constitutive α-secretase of APP in our experimental conditions (data not shown).

**Figure 6:**
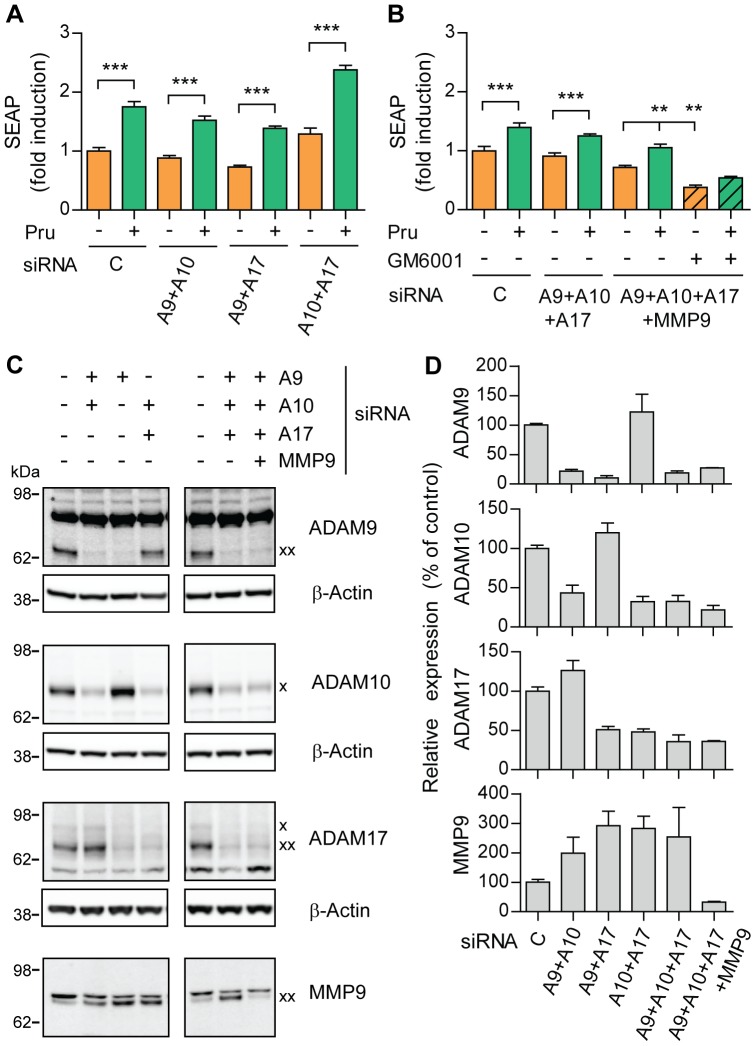
Combined knock-down of ADAM9, ADAM10, ADAM17 and MMP9 does not affect 5-HT_4d_ receptor-stimulated APP shedding. (A) and (B) SH-SY5Y cells, co-transfected with pEAK12-AP-APP, pcDNA3.1-5-HT_4_ and combinations of 3 nM siRNA for knock-down of ADAM9 (A9) and ADAM10 (A10), ADAM9 (A9) and ADAM17 (A17), ADAM10 (A10) and ADAM17 (A17) in (A) or ADAM9, 10 and 17 or ADAM9, 10, 17 and MMP9 in (B), were treated with 1 µM prucalopride (5-HT_4_ receptor agonist) in the absence or presence of 80 µM GM6001 (metalloproteinases inhibitor) (B) and secretion of sAPPα was analyzed via measuring SEAP. (C) Cell lysates of experiments in (A) and (B) were analyzed for protein expression of ADAM9, 10, 17 and MMP9 by western blotting. ADAM10 and ADAM17 immature precursor proteins are indicated by an x, whereas the mature catalytically active forms are indicated by an xx for ADAM9, 17 and MMP9. The immature ADAM9 and the mature ADAM10 proteins were not visible. (D) Quantification of experiments in (C). Values shown are mean ± SEM of 6 individual wells and were normalized to vehicle control. ** *P*<0.01, *** *P*<0.001, one-way ANOVA with Tukey-Kramer's post-hoc test.

Metalloproteinases are notorious for their functional redundancy between family members. To test whether more than one candidate metalloproteinase could be responsible for induction of α-secretase activity, we treated transfected SH-SY5Y cells with combinations of RNAi directed at ADAM9 and 10, ADAM9 and 17, ADAM10 and 17 (Figure 6A). We observed no change in sAPPα secretion upon 5-HT_4d_ receptor stimulation under any of these conditions. Moreover, knock-down of all four candidate metalloproteinases, i.e. ADAM9, 10, 17 and MMP9, still resulted in induction of sAPPα release after 5-HT_4d_ receptor activation (Figure 6B). The levels of C-terminal fragments generated by the cleavage of APP at β- and β′-sites remained unchanged after the knock-down of ADAM9, 10, 17 and MMP9, suggesting that β-secretase activity was not affected by reduced expression levels of these metalloproteinases (Figures S5A and B). We used western blotting to confirm the efficiency and specificity of RNAi mediated downregulation (Figures 6C and D). Notice also the strong upregulation of MMP9 expression when ADAM9, 10 and 17 are downregulated, while single MMP9 knock-down did not affect 5-HT_4d_ receptor-induced sAPPα secretion. Altogether, our data suggest that an unidentified GM6001-sensitive metalloproteinase participates in the regulated cleavage of APP upon 5-HT_4d_ receptor stimulation (Figure 6B).

## Discussion

**Figure 7:**
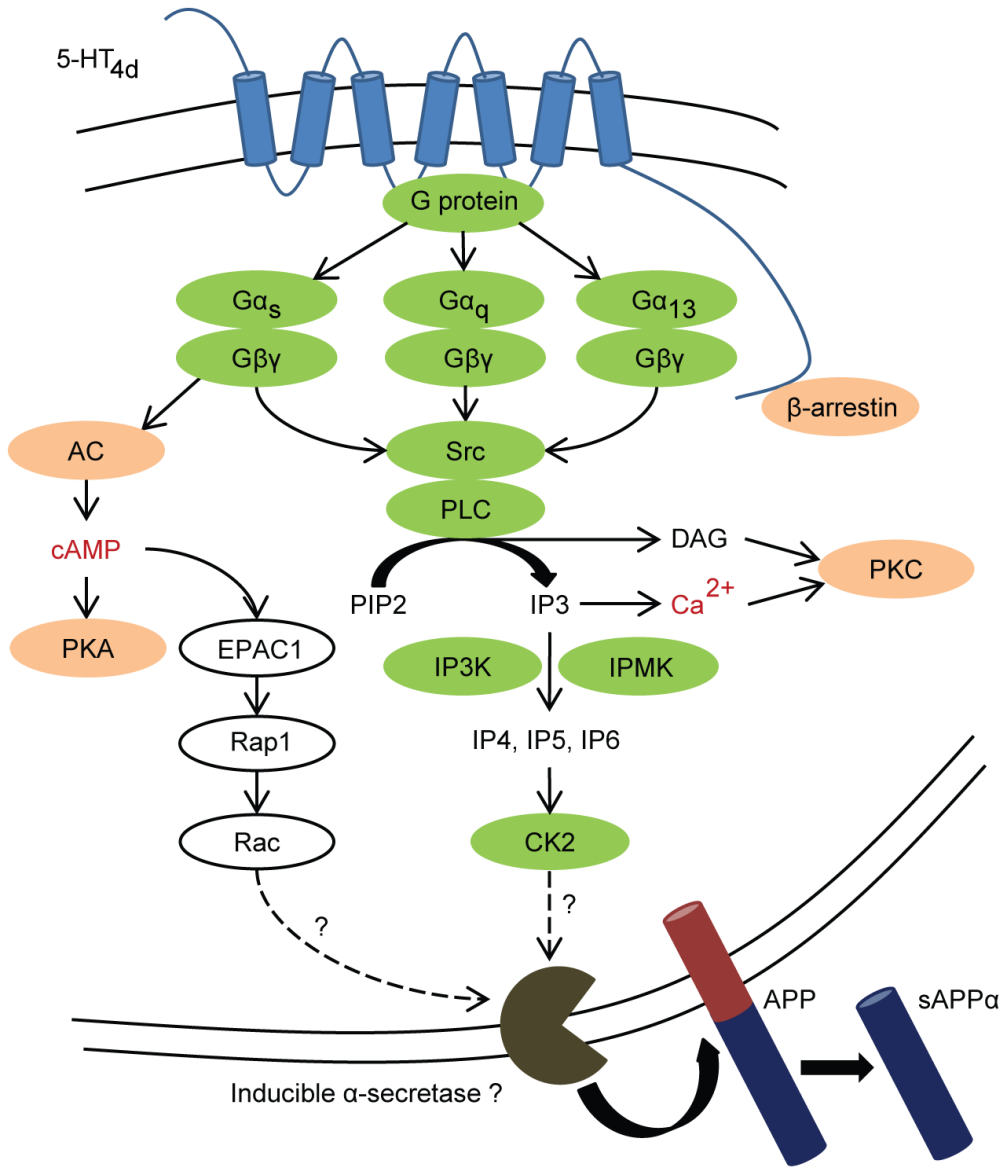
Schematic representation of the proposed 5-HT_4d_ receptor-stimulated signaling pathway leading to increased sAPPα production. The proteins involved in 5-HT_4d_ receptor-mediated non-amyloidogenic APP shedding are shown with green circles while orange circles and red characters indicate proteins or second messengers that were tested but were ineffective in modulating 5-HT_4d_ receptor-stimulated sAPPα release. The dotted lines with the question marks indicate remaining areas of investigation for further elucidation of the molecular mechanism of α-secretase activation. cAMP-dependent pathway of α-secretase induction was previously reported and is depicted as a plausible way for 5-HT_4d_ receptor-mediated sAPPα release [15].

In this report, we examined the signaling pathway that leads to α-secretase induction after 5-HT_4d_ receptor stimulation in the human neuroblastoma SH-SY5Y cell line. We present here a previously uncharacterized signaling pathway involved in the mediation of 5-HT_4d_ receptor-induced α-secretase activity (Figure 7). The characterization of this pathway was based on a combination of pharmacological, siRNA and site-directed mutagenesis experiments. Our data indicate that PLC is essential for α-secretase activation following 5-HT_4d_ stimulation. This effect is dependent on Gα and Gβγ recruitment and signaling downstream of the 5-HT_4d_ receptor. Src tyrosine kinase acts as an intermediate molecule, mediating PLC activation and inositol triphosphate production. The latter is converted by multiple kinases to inositol polyphosphates, which activate CK2. Downstream of CK2, a yet unknown mechanism of α-secretase activation is triggered. The 5-HT_4d_ receptor-induced α-secretase activity could not be ascribed to any known candidate α-secretase (ADAM9, 10, 17 and MMP9) in the SH-SY5Y cells, which has hampered delineation of the final step regulating 5-HT_4d_ receptor-stimulated sAPPα release.

We found also that β-arrestin signaling did not contribute to α-secretase activity upon 5-HT_4d_ receptor stimulation as the mutant receptor deficient in β-arrestin recruitment maintained the ability to stimulate sAPPα secretion after agonist treatment (Figure 1B). Interestingly, β-arrestins have recently emerged as regulators of Aβ generation downstream of the β2-adrenergic receptor and GPR3, independently of G protein activation [44], [45]. In these studies, β-arrestins appear to bind to the Aph1 subunit of the γ-secretase complex, affecting complex localization and thereby increasing the catalytic activity of the γ-secretase complex. Our work suggests that different signaling pathways regulate α- and γ-secretase activity as we find that G proteins are indispensable for 5-HT_4d_ receptor-stimulated α-secretase activity, while they are not involved in the γ-secretase regulation by GPCRs. Indeed, several molecules that are activated downstream of G proteins are proposed to regulate α-site APP processing, e.g. PKC, PKA, MAPK, ERK and PI3K (reviewed in [46]).

Originally the 5-HT_4_ receptor was shown to couple to Gα_s_ [17], [33]–[35], [47]. We show here that the Gα_s_, Gα_q_ and Gα_13_ subunits are equally required for sustainable α-secretase activity after 5-HT_4d_ receptor stimulation (Figure S1A). We did not observe functional compensation between the different Gα subunits, even though protein expression was modulated in a reciprocal manner for Gα_s_ and Gα_q_ (Figure S2). These results were unexpected and we speculate that parallel signaling initiated by the different Gα subunits or a certain threshold level of G proteins would be required to transduce the agonist-dependent signal. In those views reduction of the level of one of subunit could already abolish the signal. Our results also show that the signal relies on the association with the Gβγ subunits as a converging signal transduction mediator (Figure 1F). Similar observations have been previously made for PLC activation by G proteins *in vitro*. IP3 production was more efficient in the presence of the G protein trimeric complex than in separate preparations of either Gα_q_ or Gβγ alone [48], [49]. Interestingly, we also observe a reduction in constitutive sAPPα release after the knock-down of Gα_s_, Gα_q_ and Gα_13_ (Figure S1A). These data suggest a dominant negative effect of G proteins inhibition on constitutive α-secretase activity, which may be mediated by additional GPCRs besides the 5-HT_4d_ receptor.

5-HT_4_ receptor coupling to different G proteins suggests several possibilities for downstream signal transduction. Several reports describe a PKA-independent and cAMP-dependent α-secretase activation following 5-HT_4d_ receptor stimulation [7], [50], [51]. In CHO cells, sAPPα release is regulated by Epac1, which promotes small GTPases Rap1 dependent Rac activation [15]. However, we find that AC and cAMP accumulation are not required for 5-HT_4d_ receptor-induced APP shedding under our experimental conditions (Figure 2). Differences in the cellular systems, a human neuronal cell line versus a Chinese hamster ovary cell line, could explain the discrepancy between the studies. We then found that IP3 generation through Src and PLC activation contributes to 5-HT_4d_ receptor-induced α-secretase activity (Figure 3). PLC is also an important component of the α-secretase activation pathway through Gα_q_ coupled GPCRs, e.g. mGluR1 and mGluR5 [52], M1 and M3 [53], 5-HT_2a_ and 5-HT_2c_ [54] and thus a point of convergence for several transduction pathways activating α-secretase.

Investigations of the cerebral cortex and cerebellum of AD-affected individuals reveal disturbed G protein signal transduction compared to control patients [55]. In accordance, the phosphoinositide hydrolysis pathway is also altered in AD because of reduced levels of phosphatidylinositide 3-kinase and disturbed agonist and G protein regulation of PLC [56], [57]. It is proposed that 5-HT_4_ receptor stimulation could counteract such detrimental changes. We show here that the 5-HT_4d_ receptor indeed induces IP3K and IPMK mediated IP3 conversion to inositol polyphosphates and that these contribute to the non-amyloidogenic pathway of APP processing (Figure 4). This effect is mediated through the activation of CK2, which was recently identified to be downstream of the cholinergic receptors in a pathway of α-secretase induction [58]. As activation of the 5-HT_4_ receptor can increase acetylcholine levels in the brain [6], [13] and we need 24 hours to obtain a significant induction of the α-secretase, an indirect mechanism through upregulation of acetylcholine could play a role. As our cells are of the dopaminergic origin, we think this possibility is rather unlikely. However, we cannot rule out that other indirect mechanisms are playing a role in the 5-HT_4d_ receptor-mediated α-secretase induction.

To understand the molecular mechanism of α-secretase activation downstream of the 5-HT_4d_ receptor, we investigated the contribution of ADAM9, 10, 17 and MMP9 in the regulation of APP processing. Previously, regulated α-secretase activity was partially attributed to MMP9, whose expression levels increased after 5-HT_4d_ receptor stimulation in APP-overexpressing H4 human neuroglioma cells [59]. However, in our cellular system, expression levels of the investigated proteinases do not change (Figure S3) and specific protein downregulation suggests that a different metalloproteinase, besides the major candidate α-secretases ADAM9, 10, 17 and MMP9, contributes to 5-HT_4d_ receptor-induced sAPPα release (Figures 5 and 6). Indeed, ADAM10 was not responsible for the 5-HT_4d_ receptor-dependent induction of sAPPα release through the cAMP/Epac pathway [21]. At this moment, we cannot rule out that the remaining protein expression of these four major α-secretases contribute to the preserved inducible α-secretase activity. Our data are consistent with the present view of different proteases contributing to regulated APP processing as previously reported for the M1 receptor, the insulin-like growth factor-1 receptor and the purinergic P2Y2 and P2X7 receptors [23], [60]–[62]. To identify the metalloproteinase(s) responsible for induced α-secretase activity we were reluctant to use differences in susceptibility to GM6001 because we were working with overexpression conditions. This would require a large-scale RNAi knock-down study but is beyond the scope of the current manuscript.

In conclusion, our studies show the complexity of α-secretase regulation upon 5-HT_4d_ receptor stimulation. Taking into consideration that receptor modulation of signaling pathways depends on the cellular context and that recombinant overexpression and RNA interference may reveal cell type specific results, a relevant physiological system should be used for the confirmation of the identified signaling pathway. Clinical trials of agonists targeting 5-HT_4_ serotonergic and M1 muscarinic receptors will provide validation of α-secretase activation as a therapeutic approach for the treatment of AD. We report here that PLC dependent production of IP3 and CK2 activation are important mediators of the 5-HT_4d_ receptor signaling that enhance the non-amyloidogenic processing of APP. These proteins can also participate in signaling downstream of muscarinic receptors, suggesting the possibility of a common pathway for α-secretase activation through GPCRs. Finally, our data will also aid with the development of 5-HT_4_ receptor agonists as therapeutics for neurodegenerative or psychiatric disorders and allow for a better understanding of potential risks associated with these drugs.

## Supporting Information

Figure S1 **5-HT_4d_ receptor-stimulated APP shedding requires the G proteins Gα_s_, Gα_q_ and Gα_13_.** (A) SEAP levels were measured in supernatants of SH-SY5Y cells, co-transfected with pEAK12-AP-APP, pcDNA3.1-5-HT_4d_ and 3 nM siRNA for knock-down of Gα_s_, Gα_q_ and Gα_13_ and treated with 1 µM prucalopride (5-HT_4_ receptor agonist). (B) Cell lysates of (A) were analyzed for protein expression of Gα_s_, Gα_q_ and Gα_13_ by western blotting. (C) Quantification of experiments in (B). Values shown are mean ± SEM of 6 individual wells and were normalized to vehicle control. ** *P*<0.01, *** *P*<0.001, one-way ANOVA with Tukey-Kramer's post-hoc test.
(TIF)

Figure S2 **Knock-down of Gα_s_ and Gα_q_ but not Gα_13_ alters protein expression of the G protein family.** (A) SH-SY5Y cells, transfected with 3 nM siRNA for knock-down of Gα_s_, Gα_q_ and Gα_13_, were harvested and expression levels of G proteins were analyzed. (B) Quantification of experiments in (A). Values shown are mean ± SEM of 2 individual wells and were normalized to vehicle control. ** *P*<0.01, one-way ANOVA with Tukey-Kramer's post-hoc test.
(TIF)

Figure S3 **Expression levels of major candidate α-secretases and APP do not change upon 5-HT_4d_ receptor stimulation.** (A) SH-SY5Y cells, transfected with pEAK12-AP-APP and pcDNA3.1-5-HT_4d_, were treated with 1 µM prucalopride (5-HT_4_ receptor agonist) and collected to analyze protein expression of ADAM9, 10, 17, MMP9 and APP by western blotting. ADAM10 and ADAM17 immature precursor proteins are indicated by an x, whereas the mature catalytically active forms are indicated by an xx for ADAM9, 17 and MMP9. The immature ADAM9 and the mature ADAM10 proteins were not visible. (B) Quantification of experiments in (A). Values shown are mean ± SEM of 2 individual wells and were normalized to vehicle control.
(TIF)

Figure S4 **The metalloproteinase inhibitor GM6001 can inhibit secretion of sAPPα upon 5-HT_4d_ receptor stimulation.** SH-SY5Y cells, transfected with pEAK12-AP-APP and pcDNA3.1-5-HT_4d_, were treated with 1 µM prucalopride or 5-HT in the absence or presence of different concentrations of GM6001 and secretion of sAPPα was analyzed via measuring SEAP. Values shown are mean ± SEM of 6 individual wells and are normalized towards vehicle control. ** *P*<0.01, *** *P*<0.001, one-way ANOVA with Tukey-Kramer's post-hoc test.
(TIF)

Figure S5 **Knock-down of ADAM9, 10, 17 and MMP9 does not affect the pattern of CTFs generated by the 5-HT_4d_ receptor-stimulated α-secretase activity.** (A) SH-SY5Y cells, co-transfected with pEAK12-AP-APP, pcDNA3.1-5-HT_4d_ and combinations of 3 nM siRNA for knock-down of ADAM9 (A9), ADAM10 (A10) and ADAM17 (A17) or ADAM9, 10, 17 and MMP9, were treated with 1 µM prucalopride (5-HT_4_ receptor agonist) and secretion of sAPPα was analyzed via measuring SEAP. Values shown are mean ± SEM of 6 individual wells and were normalized to vehicle control. *** *P*<0.001, one-way ANOVA with Tukey-Kramer's post-hoc test. (B) Cell lysates of the experiment in (A) were analyzed for the levels of different APP C-terminal fragments (CTFs) were analyzed by western blotting using B63 antibody (16% Tricine SDS-PAGE).
(TIF)

Table S1 (DOCX)
