# Supplementary figures and images for: Regulation of Amyloid Precursor Protein Processing by Serotonin Signaling
Source: PLoS One. 2014 Jan 21;9(1):e87014. doi: 10.1371/journal.pone.0087014 (PMC3897773; doi:10.1371/journal.pone.0087014)

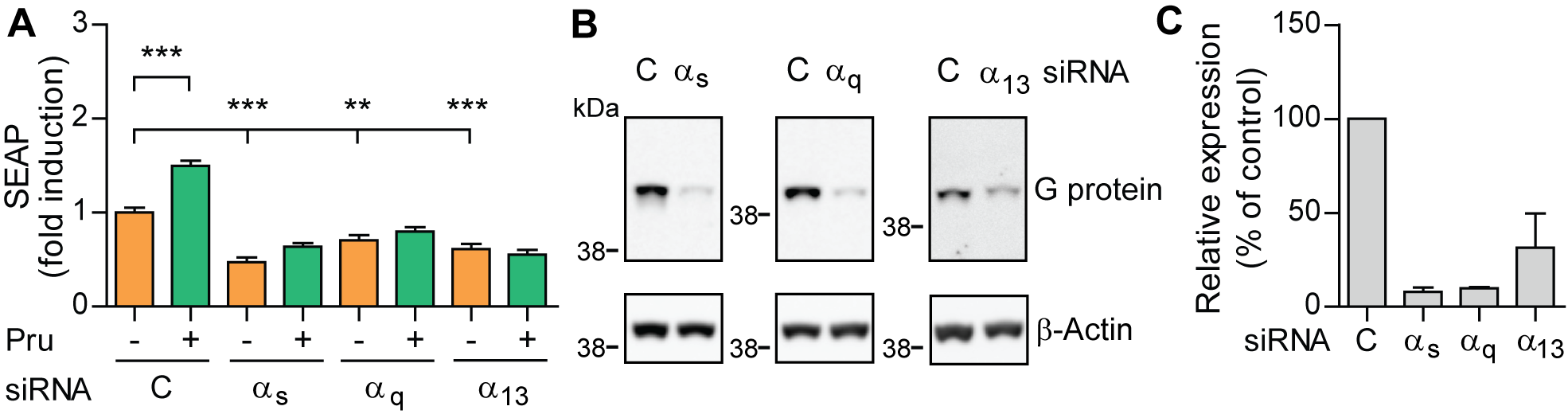

Supplement: Figure S1 — 5-HT4d receptor-stimulated APP shedding requires the G proteins Gαs, Gαq and Gα13. (A) SEAP levels were measured in supernatants of SH-SY5Y cells, co-transfected with pEAK12-AP-APP, pcDNA3.1-5-HT4d and 3 nM siRNA for knock-down of Gαs, Gαq and Gα13 and treated with 1 µM prucalopride (5-HT4 receptor agonist). (B) Cell lysates of (A) were analyzed for protein expression of Gαs, Gαq and Gα13 by western blotting. (C) Quantification of experiments in (B). Values shown are mean ± SEM of 6 individual wells and were normalized to vehicle control. ** P<0.01, *** P<0.001, one-way ANOVA with Tukey-Kramer's post-hoc test. (TIF) [file pone.0087014.s001.tif]

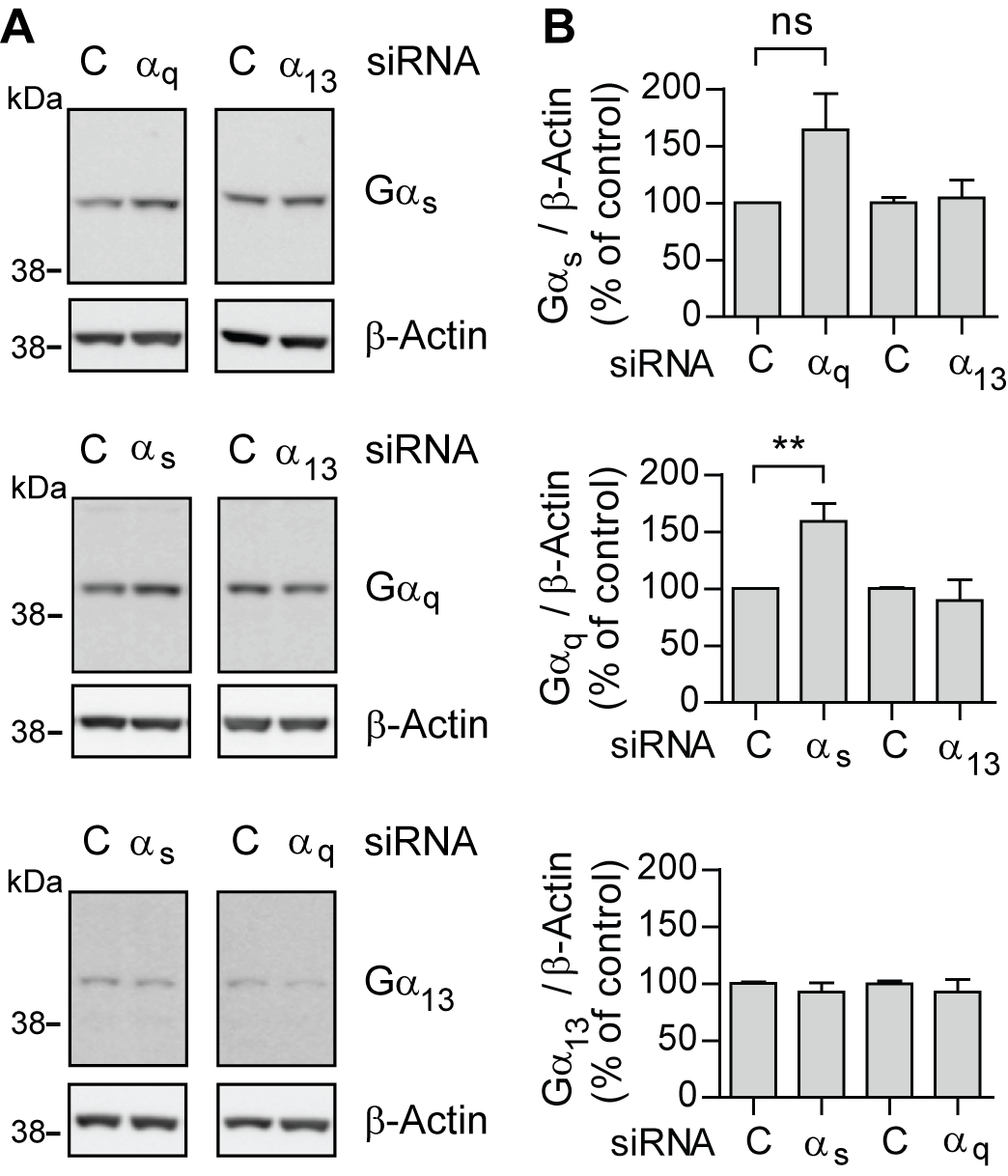

Supplement: Figure S2 — Knock-down of Gαs and Gαq but not Gα13 alters protein expression of the G protein family. (A) SH-SY5Y cells, transfected with 3 nM siRNA for knock-down of Gαs, Gαq and Gα13, were harvested and expression levels of G proteins were analyzed. (B) Quantification of experiments in (A). Values shown are mean ± SEM of 2 individual wells and were normalized to vehicle control. ** P<0.01, one-way ANOVA with Tukey-Kramer's post-hoc test. (TIF) [file pone.0087014.s002.tif]

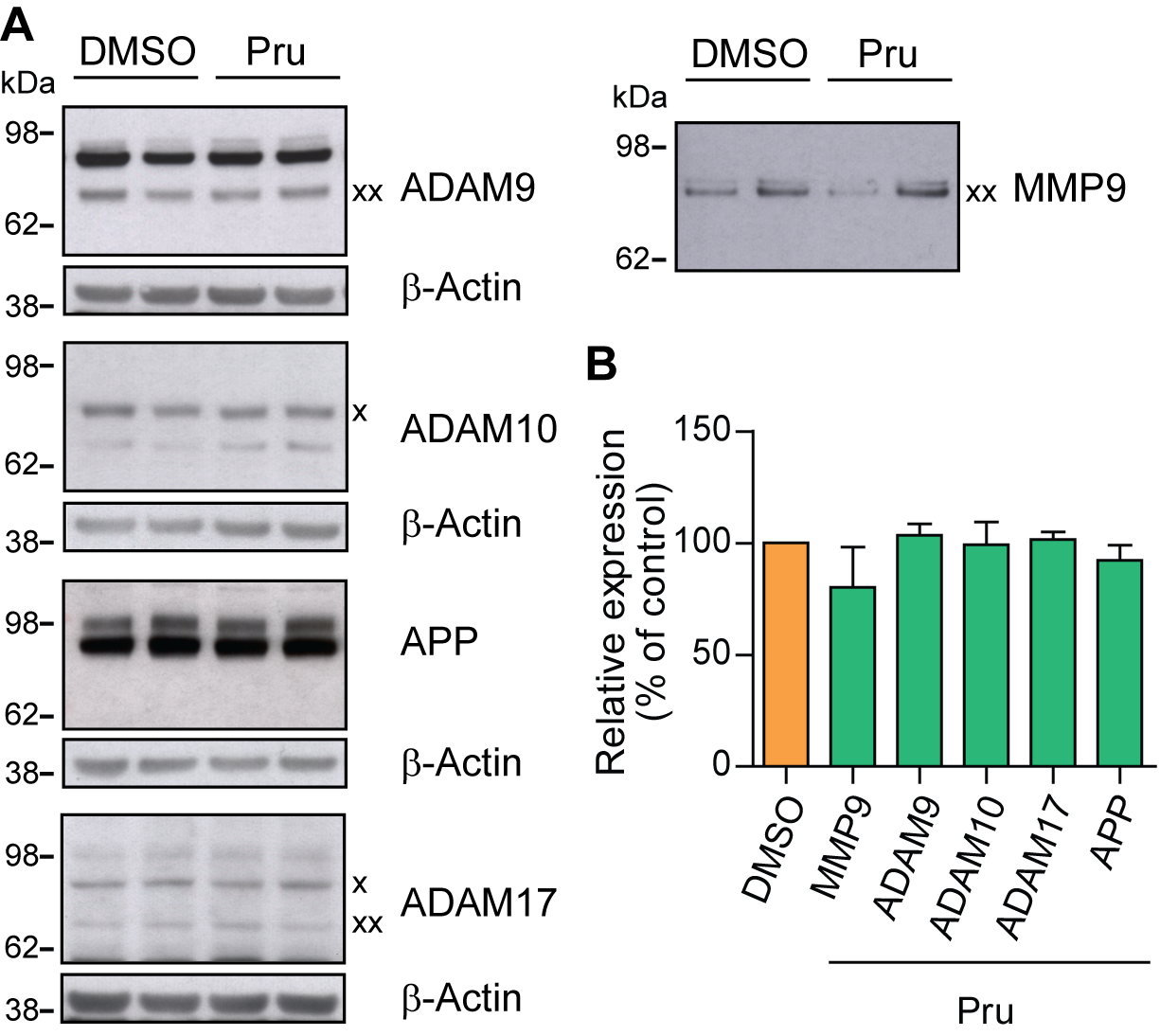

Supplement: Figure S3 — Expression levels of major candidate α-secretases and APP do not change upon 5-HT4d receptor stimulation. (A) SH-SY5Y cells, transfected with pEAK12-AP-APP and pcDNA3.1-5-HT4d, were treated with 1 µM prucalopride (5-HT4 receptor agonist) and collected to analyze protein expression of ADAM9, 10, 17, MMP9 and APP by western blotting. ADAM10 and ADAM17 immature precursor proteins are indicated by an x, whereas the mature catalytically active forms are indicated by an xx for ADAM9, 17 and MMP9. The immature ADAM9 and the mature ADAM10 proteins were not visible. (B) Quantification of experiments in (A). Values shown are mean ± SEM of 2 individual wells and were normalized to vehicle control. (TIF) [file pone.0087014.s003.tif]

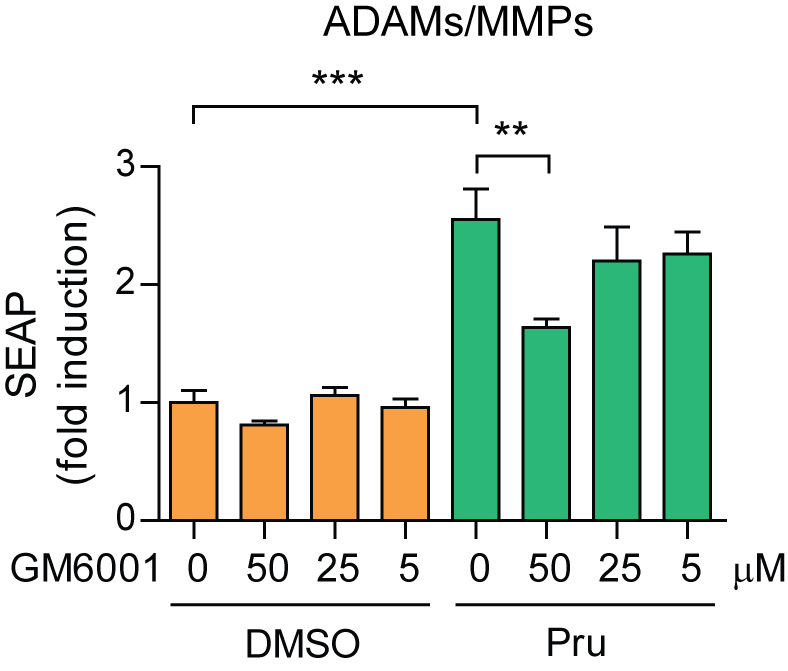

Supplement: Figure S4 — The metalloproteinase inhibitor GM6001 can inhibit secretion of sAPPα upon 5-HT4d receptor stimulation. SH-SY5Y cells, transfected with pEAK12-AP-APP and pcDNA3.1-5-HT4d, were treated with 1 µM prucalopride or 5-HT in the absence or presence of different concentrations of GM6001 and secretion of sAPPα was analyzed via measuring SEAP. Values shown are mean ± SEM of 6 individual wells and are normalized towards vehicle control. ** P<0.01, *** P<0.001, one-way ANOVA with Tukey-Kramer's post-hoc test. (TIF) [file pone.0087014.s004.tif]

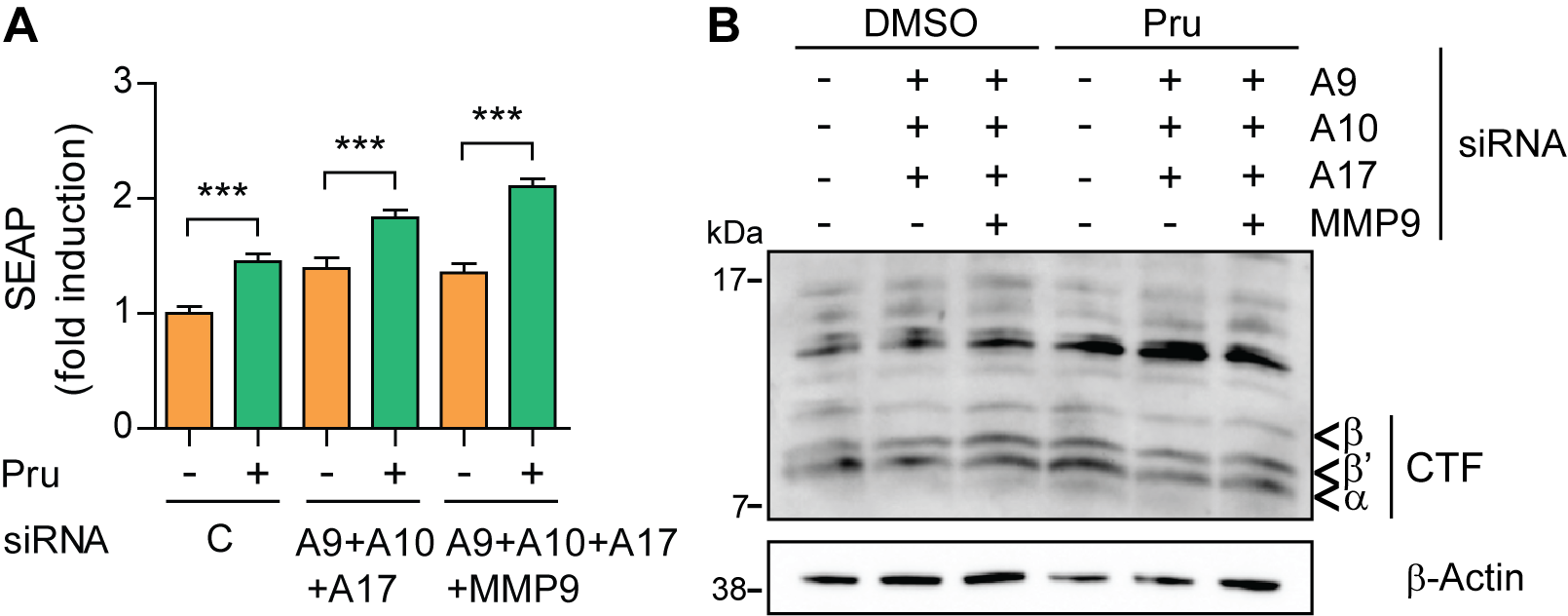

Supplement: Figure S5 — Knock-down of ADAM9, 10, 17 and MMP9 does not affect the pattern of CTFs generated by the 5-HT4d receptor-stimulated α-secretase activity. (A) SH-SY5Y cells, co-transfected with pEAK12-AP-APP, pcDNA3.1-5-HT4d and combinations of 3 nM siRNA for knock-down of ADAM9 (A9), ADAM10 (A10) and ADAM17 (A17) or ADAM9, 10, 17 and MMP9, were treated with 1 µM prucalopride (5-HT4 receptor agonist) and secretion of sAPPα was analyzed via measuring SEAP. Values shown are mean ± SEM of 6 individual wells and were normalized to vehicle control. *** P<0.001, one-way ANOVA with Tukey-Kramer's post-hoc test. (B) Cell lysates of the experiment in (A) were analyzed for the levels of different APP C-terminal fragments (CTFs) were analyzed by western blotting using B63 antibody (16% Tricine SDS-PAGE). (TIF) [file pone.0087014.s005.tif]
