# Supplementary material for: Regulation of Amyloid Precursor Protein Processing by Serotonin Signaling
Source: PLoS One. 2014 Jan 21;9(1):e87014. doi: 10.1371/journal.pone.0087014 (PMC3897773; doi:10.1371/journal.pone.0087014)
Supplement: Table S1 — (DOCX) [file pone.0087014.s006.docx]

# SUPPORTING INFORMATION

**Table S1. Overview of agonists, antagonists and inhibitors used to investigate the proteins contributing to the induction of sAPPα after 5-HT_4d_ receptor stimulation.**

| Compound | Target | Ag / antag / inh | Potency | Experimental system | Citation |
| --- | --- | --- | --- | --- | --- |
| Prucalopride | 5-HT_4_ | Ag | EC_50_ 10 nM | SH-SY5Y cells | [[1](#_ENREF_1)] |
| 5-HT | 5-HT_4_ | Ag | EC_50_ 1,1 nM | HEK293 cells | [[2](#_ENREF_2)] |
| GR113808 | 5-HT_4_ | Antag | K_i_ 0,31 nM | Mouse colliculi neurons | [[3](#_ENREF_3),[4](#_ENREF_4)] |
| Cholera toxin B (CTB) | Gα_s_ | Inh | IC_50_ 100 ng/ml | L6 cells | [[5](#_ENREF_5),[6](#_ENREF_6)] |
| NF449 | Gα_s_ | Inh | IC_50_ 8 µM | *in vitro* | [[7](#_ENREF_7)] |
| Gallein | Gβγ | Inh | IC_50_ 5 µM | HL60 cells | [[8](#_ENREF_8)] |
| SQ 22536 | Adenylyl cyclase | Inh | IC_50_ 1 µM | Human blood platelets | [[9](#_ENREF_9),[10](#_ENREF_10)] |
| 2,5-dideoxyadenosine (DDA) | Adenylyl cyclase | Inh | IC_50_ 100 µM | *in vitro* | [[11](#_ENREF_11),[12](#_ENREF_12)] |
| Bosutinib | Src | Inh | IC_50_ 300 nM | MDA-MB-468 cells | [[13](#_ENREF_13),[14](#_ENREF_14)] |
| D609 | Phospholipase C | Inh | K_i_ 6,4 µM | *in vitro* | [[15](#_ENREF_15),[16](#_ENREF_16)] |
| GF109203X | Protein kinase C | Inh | IC_50_ ≤ 5,8 µM | *in vitro* | [[17](#_ENREF_17),[18](#_ENREF_18)] |
| IP3K inhibitor | IP6K, IP3K | Inh | IC_50_ 18 µM | *in vitro* | [[19](#_ENREF_19)] |
| Chlorogenic acid (CGA) | IPMK | Inh | IC_50_ 1,15 µM | *in vitro* | [[20](#_ENREF_20)] |
| 4,5,6,7-tetrabromo-1H-benzotriazole (TBB) | Casein kinase 2 | Inh | IC_50_ 1,6 μM | *in vitro* | [[21-23](#_ENREF_21)] |
| GM6001 | MMP1, 2, 3, 8, 9; ADAM10 and 17 | Inh | K_i_ 0,1–110 nM | *in vitro* | [[24](#_ENREF_24),[25](#_ENREF_25)] |

Ag = agonist; antag = antagonist; inh = inhibitor.

1. Tesseur I, Pimenova AA, Lo AC, Ciesielska M, Lichtenthaler SF, et al. (2013) Chronic 5-HT4 receptor activation decreases Abeta production and deposition in hAPP/PS1 mice. Neurobiol Aging 34: 1779-1789.

2. Chang WC, Ng JK, Nguyen T, Pellissier L, Claeysen S, et al. (2007) Modifying ligand-induced and constitutive signaling of the human 5-HT4 receptor. PLoS One 2: e1317.

3. Ansanay H, Sebben M, Bockaert J, Dumuis A (1996) Pharmacological comparison between [3H]GR 113808 binding sites and functional 5-HT4 receptors in neurons. Eur J Pharmacol 298: 165-174.

4. Van den Wyngaert I, Gommeren W, Verhasselt P, Jurzak M, Leysen J, et al. (1997) Cloning and expression of a human serotonin 5-HT4 receptor cDNA. J Neurochem 69: 1810-1819.

5. Milligan G, Unson CG, Wakelam MJ (1989) Cholera toxin treatment produces down-regulation of the alpha-subunit of the stimulatory guanine-nucleotide-binding protein (Gs). Biochem J 262: 643-649.

6. Shen KF, Crain SM (1990) Cholera toxin-B subunit blocks excitatory effects of opioids on sensory neuron action potentials indicating that GM1 ganglioside may regulate Gs-linked opioid receptor functions. Brain Res 531: 1-7.

7. Hohenegger M, Waldhoer M, Beindl W, Boing B, Kreimeyer A, et al. (1998) Gsalpha-selective G protein antagonists. Proc Natl Acad Sci U S A 95: 346-351.

8. Lehmann DM, Seneviratne AM, Smrcka AV (2008) Small molecule disruption of G protein beta gamma subunit signaling inhibits neutrophil chemotaxis and inflammation. Mol Pharmacol 73: 410-418.

9. Harris DN, Asaad MM, Phillips MB, Goldenberg HJ, Antonaccio MJ (1979) Inhibition of adenylate cyclase in human blood platelets by 9-substituted adenine derivatives. J Cyclic Nucleotide Res 5: 125-134.

10. Gao Y, Usha Raj J (2002) Effects of SQ 22536, an adenylyl cyclase inhibitor, on isoproterenol-induced cyclic AMP elevation and relaxation in newborn ovine pulmonary veins. Eur J Pharmacol 436: 227-233.

11. Onoda JM, Braun T, Wrenn SM, Jr. (1987) Characterization of the purine-reactive site of the rat testis cytosolic adenylate cyclase. Biochem Pharmacol 36: 1907-1912.

12. Legrand AB, Narayanan TK, Ryan US, Aronstam RS, Catravas JD (1990) Effects of adenosine and analogs on adenylate cyclase activity in cultured bovine aortic endothelial cells. Biochem Pharmacol 40: 1103-1109.

13. Coluccia AM, Benati D, Dekhil H, De Filippo A, Lan C, et al. (2006) SKI-606 decreases growth and motility of colorectal cancer cells by preventing pp60(c-Src)-dependent tyrosine phosphorylation of beta-catenin and its nuclear signaling. Cancer Res 66: 2279-2286.

14. Vultur A, Buettner R, Kowolik C, Liang W, Smith D, et al. (2008) SKI-606 (bosutinib), a novel Src kinase inhibitor, suppresses migration and invasion of human breast cancer cells. Mol Cancer Ther 7: 1185-1194.

15. Amtmann E (1996) The antiviral, antitumoural xanthate D609 is a competitive inhibitor of phosphatidylcholine-specific phospholipase C. Drugs Exp Clin Res 22: 287-294.

16. Tschaikowsky K, Meisner M, Schonhuber F, Rugheimer E (1994) Induction of nitric oxide synthase activity in phagocytic cells inhibited by tricyclodecan-9-yl-xanthogenate (D609). Br J Pharmacol 113: 664-668.

17. Martiny-Baron G, Kazanietz MG, Mischak H, Blumberg PM, Kochs G, et al. (1993) Selective inhibition of protein kinase C isozymes by the indolocarbazole Go 6976. J Biol Chem 268: 9194-9197.

18. Jacobson PB, Kuchera SL, Metz A, Schachtele C, Imre K, et al. (1995) Anti-inflammatory properties of Go 6850: a selective inhibitor of protein kinase C. J Pharmacol Exp Ther 275: 995-1002.

19. Chang YT, Choi G, Bae YS, Burdett M, Moon HS, et al. (2002) Purine-based inhibitors of inositol-1,4,5-trisphosphate-3-kinase. Chembiochem 3: 897-901.

20. Mayr GW, Windhorst S, Hillemeier K (2005) Antiproliferative plant and synthetic polyphenolics are specific inhibitors of vertebrate inositol-1,4,5-trisphosphate 3-kinases and inositol polyphosphate multikinase. J Biol Chem 280: 13229-13240.

21. Sarno S, Reddy H, Meggio F, Ruzzene M, Davies SP, et al. (2001) Selectivity of 4,5,6,7-tetrabromobenzotriazole, an ATP site-directed inhibitor of protein kinase CK2 ('casein kinase-2'). FEBS Lett 496: 44-48.

22. Pagano MA, Andrzejewska M, Ruzzene M, Sarno S, Cesaro L, et al. (2004) Optimization of protein kinase CK2 inhibitors derived from 4,5,6,7-tetrabromobenzimidazole. J Med Chem 47: 6239-6247.

23. Zien P, Duncan JS, Skierski J, Bretner M, Litchfield DW, et al. (2005) Tetrabromobenzotriazole (TBBt) and tetrabromobenzimidazole (TBBz) as selective inhibitors of protein kinase CK2: evaluation of their effects on cells and different molecular forms of human CK2. Biochim Biophys Acta 1754: 271-280.

24. Galardy RE, Cassabonne ME, Giese C, Gilbert JH, Lapierre F, et al. (1994) Low molecular weight inhibitors in corneal ulceration. Ann N Y Acad Sci 732: 315-323.

25. Moss ML, Rasmussen FH (2007) Fluorescent substrates for the proteinases ADAM17, ADAM10, ADAM8, and ADAM12 useful for high-throughput inhibitor screening. Anal Biochem 366: 144-148.
